# Photosensitive Dye as an Ideal Peroxymonosulfate Activator for Efficient Self-Degradation: A Novel Idea of Using Waste to Treat Waste

**DOI:** 10.3390/molecules28104237

**Published:** 2023-05-22

**Authors:** Zhiyao Zhang, Zhaolin Li, Xue Bai, Juan Shi, Min Hu, Jin Chai, Keqian Li, Pengkang Jin

**Affiliations:** 1 School of Environmental and Municipal Engineering, Xi’an University of Architecture and Technology, Xi’an 710055, China; 2 Oil and Gas Technology Research Institute, Petrochina Changqing Oilfield Company, Xi’an 710018, China; 3 School of Human Settlements and Civil Engineering, Xi’an Jiaotong University, Xi’an 710049, China

**Keywords:** alizarin green dye, photosensitization, PMS, catalysis, using waste to treat waste

## Abstract

Commonly used peroxydisulfate (PS) or peroxymonosulfate (PMS) activation methods have been limited in their practical application due to certain drawbacks, such as high cost, high energy consumption and secondary pollution. In this study, a catalyst-free alizarin green (AG) self-activating PMS catalytic system was constructed based on photosensitization properties of dye, which ultimately achieved efficient degradation of the dye activator, also the target pollutant. Here, 52.5% of the 100 mL mixture of 10 mg/L AG decomposed within 60 min with 1 mM PMS under visible-light irradiation, thereby showing a strong pH adaptation. Mechanism of AG self-activating PMS was revealed that the photo-excited AG can effectively transfer photo-induced electrons to PMS for its activation, which generates reactive oxidizing species dominated by singlet oxygen (^1^O_2_), and supplemented by hydroxyl radical (•OH), superoxide radical (O_2_^•−^) and sulfate radical (SO_4_^•−^) to realize the efficient self-degradation of the dye pollutants. Moreover, such self-catalytic system operated well under natural sunlight irradiation, indicating the great application potential in the actual wastewater treatment. Herein, photosensitive dye acted as an ideal PMS activator realizing its efficient self-degradation, which provides a novel idea of “using waste to treat waste” for developing wastewater treatment process in a high-efficiency and low-consumption way.

## 1. Introduction

With the acceleration of industrialization and urbanization, environmental problems, such as water pollution are rapidly aggravated. Textile, food, paper and leather related industries use large amounts of dyes [1,2], of which about 10–15% are discharged with the effluent [3]. Furthermore, the color removal and complete mineralization of dyeing wastewater face great challenges due to the complex structure of dye molecules [4,5,6]. Nowadays, with the expansion of the banned range of azo dyes, the importance of anthraquinone dyes has become more prominent. Since the benzene ring on both sides of the parent anthraquinone dye is affected by two carbonyl groups at the same time, the structures of anthraquinone dyes are complex and stable, and even have a certain potential toxicity [7]. Advanced oxidation processes (AOPs) centered on strong oxidizing radicals represented by hydroxyl radical (•OH) have attracted much attention in the treatment of refractory pollutants [8,9,10,11]. Recently, the emerging sulfate radical (SO_4_^•−^) advanced oxidation processes (SR-AOPs) have become a promising approach due to their stronger oxidative properties, longer half-life and broader pH applicability relative to •OH [12,13,14,15,16], which is initiated by persulfate activation, specifically peroxymonosulfate (PMS) and peroxydisulfate (PS). PMS or PS activation is commonly realized via heating, alkali, electricity, radiation, homogeneous transition metal ion and heterogeneous catalyst, however, all of these routes require large amounts of energy input or various catalysts addition, resulting in high costs, high energy consumption and secondary pollution [8,17,18]. Therefore, novel methods for PMS or PS activation are desired for economical and sustainable wastewater treatment processes [19].

Recent studies showed that persulfate could be activated by some organics without external energy input and catalyst addition. For instance, both PS and PMS could be activated by benzoquinone, but the mechanisms greatly differ; while PS was activated through a Fenton-like reaction that required a semiquinone radical [20], the activation of PMS followed a dioxirane intermediate pathway [21]. Ahmad et al. discovered that PS was activated by ionic phenol through a single-electron reduction process [17].

(1) $${\mathrm{PhO}}^{-}+{\;S}_{2}O_{8}{}^{2-}\;\to {\;\mathrm{SO}}_{4}{}^{2-}+{\;\mathrm{SO}}_{4}{}^{•-}+{\mathrm{PhO}}_{\mathrm{ox}}$$

Richard et al. discovered that PS could be activated by glucose at minimal concentrations (1~5 mM), and the mechanism was likely that of ionic phenols, meanwhile, the products of glucose oxidation might also participate in the subsequent activation processes [22].

(2) $$\mathrm{glucose}\;+{\;S}_{2}O_{8}{}^{2-}\;\to {\;\mathrm{SO}}_{4}{}^{2-}+{\;\mathrm{SO}}_{4}{}^{•-}+\mathrm{products}$$

Additionally, Hou et al. revealed that ascorbic acid (AA) could also activate PS via the enediol group, in which electrons were transferred from ascorbic acid to PS, causing a two-step oxidation of ascorbic acid and the production of SO_4_^•−^ [23]. The reported persulfate activation processes indicate that some organic substances can directly activate persulfate through an efficient electron transfer process.

Previously, our research group designed several kinds of dye-sensitized photocatalysts, in which organic dyes were combined with semiconductors [24,25]. Then, the corresponding catalytic system for persulfate activation was constructed, where the electrons from photo-excited dye can be transferred to the complexed semiconductor and further be captured by persulfate generating a variety of reactive oxidizing species to take part in the pollutants degradation process [25]. Based on this study and considering the above-mentioned reports of persulfate activation by organics, we proposed a new idea, i.e., whether the excited photosensitive dyes can directly transfer electrons to persulfate in the absence of a semiconductor. If the thought can be realized, it will provide a novel way of “using waste to treat waste” for dyeing wastewater purification, where the photosensitive dyes self-activate persulfate without additional catalyst producing reactive oxidizing species for efficient degradation of the dye itself, thus building an eco-friendly, economically feasible and sustainable way for pollutant degradation.

For the purpose of exploring the feasibility of the proposed self-catalytic route, in this study, we constructed a reaction system of photosensitive dye self-activating PMS under visible-light irradiation, in which anthraquinone dye alizarin green (AG) was employed as the PMS activator as well as the targeted pollutant, and the reaction efficiency was evaluated based on the degradation ratio of the dye. On this basis, the main factors affecting the reaction process were investigated separately, meanwhile, the mechanism of reaction in this system was explored according to the identification of the reactive oxidizing species, the characterization of the electron transfer process and the degradation route of photosensitive dye. Moreover, we conducted a preliminary practical application of the designed catalytic system. The research can not only propose a novel idea of “using waste to treat waste” pathway for dyeing wastewater purification, but also provide the theoretical support to develop SR-AOPs in a high-efficiency and low-consumption way.

## 2. Results and Discussion

### 2.1. Performance of AG/PMS Self-Catalytic System 

**Figure 1:**
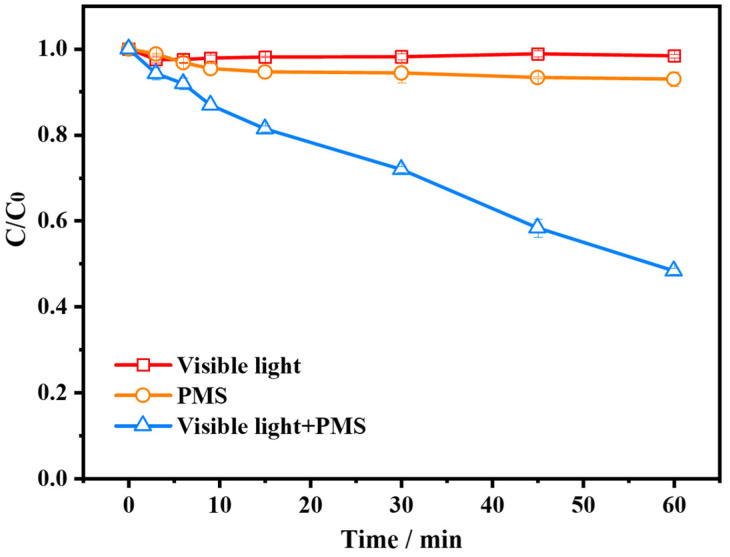
Degradation efficiency of AG (alizarin green) in different reaction systems.

In order to explore the feasibility of the proposed self-catalytic route, 10 mg/L AG was employed as a PMS activator and a targeted contaminant simultaneously. As shown in Figure 1, there was negligible degradation of AG when only visible light was available, which indicates that the dye cannot be directly photolyzed. When only PMS existed, the degradation ratio of AG was 7.0%, this may be owing to the certain oxidation capacity of PMS which can degrade a little amount of AG molecules [26]. Surprisingly, when both visible light and PMS were present, the degradation ratio of AG greatly increased to 52.2%, meanwhile the PMS activation efficiency was 20.3%. This phenomenon may be attributed to the fact that photo-excited AG dye is able to interact with PMS producing a variety of reactive oxidizing species, thus facilitating the decomposition process of the dye itself.

### 2.2. Effects of Different Parameters on AG/PMS Self-Catalytic System 

#### 2.2.1. Initial AG Concentration

**Figure 2:**
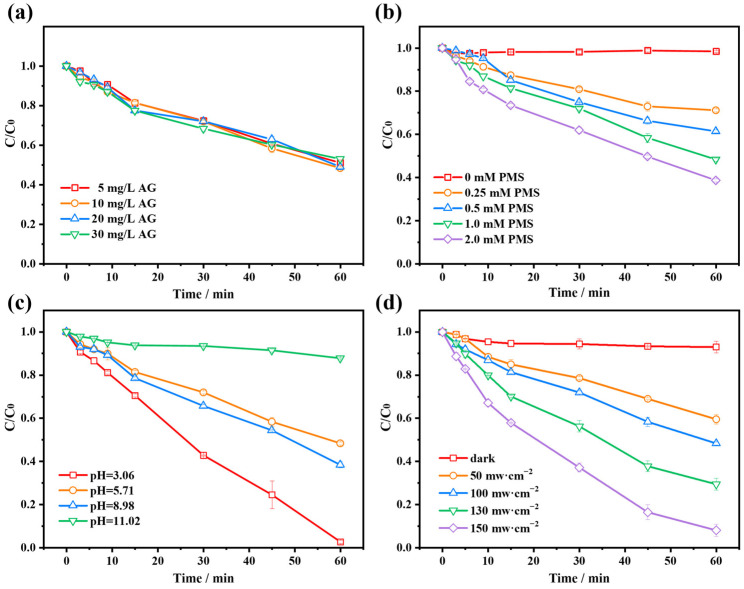
Effects of different (**a**) AG concentrations; (**b**) PMS (peroxymonosulfate) dosage; (**c**) initial solution pH; (**d**) light intensity on the degradation efficiency of AG in AG/PMS self-catalytic system.

Effect of different dye concentrations on the AG/PMS self-catalytic system is displayed in Figure 2a. The degradation ratio of different concentrations of AG all remained around 50% almost without a difference. Under light irradiation and when PMS dosage is fixed, a higher concentration of the photosensitive dye contributes to producing much more photo-induced electrons, which may be captured by the PMS and generates several kinds of reactive oxidizing species for dye removal [4]. However, in this reaction system, AG is not only the PMS activator but also the targeted pollutant, therefore the larger initial concentration of the dye implies a large quantity of pollutant molecules, which will influence the final efficiency of pollutant removal. Hence, comprehensively considering the balance between the generated reactive oxidizing species and pollutant molecules, the variations of dye concentration in a certain range have no significant influence on its self-catalytic system.

#### 2.2.2. PMS Dosage

Effect of different oxidant dosages on the AG/PMS self-catalytic system is shown in Figure 2b. A negligible degradation of AG is observed without PMS, indicating that the dye cannot be directly photolyzed. As the dosage of PMS is raised from 0.25 to 2.0 mM, the AG degradation ratio increased from 30% to 63%. Therefore, it is evident that a higher concentration of PMS contributes to improving the degradation efficiency of the dye. When the dye concentration is fixed, a higher PMS dosage benefits the collision probability between the two reactants, which may promote the photo-induced electrons transfer from the dye to PMS, consequently generating much more reactive oxidizing species for efficient pollutant degradation [27].

#### 2.2.3. Initial Solution pH

In general, pH plays a significant role in AOPs because it influences the forms of organic compounds and the types of reactive oxidizing species [12]. Effect of different solution pH on the AG/PMS self-catalytic system is presented in Figure 2c. The initial pH of the aqueous solution containing AG and PMS is 7.33. With the increase in initial pH from 3.06 to 5.71, the degradation ratio of AG decreased from 96% to 53%, then slightly increased to 61% when the pH reached 8.98. However, the degradation ratio sharply decreased to 13% when pH further increased to 11.02. This phenomenon may be attributed to the change in electrostatic interaction between the different existing forms of dye molecules and PMS under different pH circumstances.

It has been demonstrated that AG is an acidic dye whose dissociation constant (pK_a_) is around 4.5 [28]. Therefore, it takes on the cationic form as pH is below 4.5, whereas it exists mainly in amphoteric form and especially in anionic form under alkaline condition. As for PMS, it has a pK_a1_ of lower than 0 and pK_a2_ of 9.4, so HSO_5_^−^ predominates in both acidic and neutral solution, while SO_5_^2−^ concentration sharply increases when the pH is higher than 9.2 [29]. When the pH was around 3.06, AG exists in cationic form and has strong coulombic attraction with HSO_5_^−^, which benefits photo-induced electrons transfer between the photo-excited dye and PMS, thus producing more reactive oxidizing species and promoting dye degradation. When the pH was 5.71, AG exists in the amphoteric form and has weaker coulombic attraction with HSO_5_^−^ relative to that of 3.06, which is not conducive to electron transfer, thus influencing the PMS activation process and decreasing the degradation ratio of the dye. When the pH was 8.98, the AG mainly exists in anions, which has electrostatic repulsion with HSO_5_^−^, nevertheless, since PMS can be activated under alkaline conditions to generate reactive oxidizing species [26,30], the degradation ratio of AG at pH 8.98 was slightly higher than that at 5.71. When the pH further increased to 11.02, AG exists mainly in the form of anion and has a stronger repulsive effect with SO_5_^2−^ than HSO_5_^−^ at pH 8.98, which hinders the electron transfer process, thus leading to a poor yield of reactive oxidizing species generation and low efficiency of pollutant degradation [31]. According to the analysis above, we can conclude that the AG/PMS self-catalytic system operated well under acidic, neutral and alkaline conditions, indicating that the proposed catalytic route is of practical application potential due to the wide pH adaptability.

#### 2.2.4. Light Intensity

Figure 2d depicts the effect of various light intensities on the AG/PMS self-catalytic system. The degradation ratio of AG was 7% without light irradiation, which is due to the oxidation ability of PMS itself. The dye degradation ratio increased from 40.5% to 91.9% as the visible light intensity raised from 50 to 150 mW·cm^−2^. This is because the light intensity significantly affects the photo-induced electron yield of photosensitive dyes. In general, at a certain dye concentration, if the irradiation light intensity is stronger, then more charge carriers will be generated [25]. Thereafter, the increased photo-induced electrons are delivered to PMS, which can significantly enhance the activation efficiency of PMS, thus producing more reactive oxidizing species involved in the pollutant degradation [32]. Therefore, there is a substantial increase in dye degradation ratio in response to the rise of light intensity.

### 2.3. Application of AG/PMS Self-Catalytic System under Natural Sunlight Irradiation

**Figure 3:**
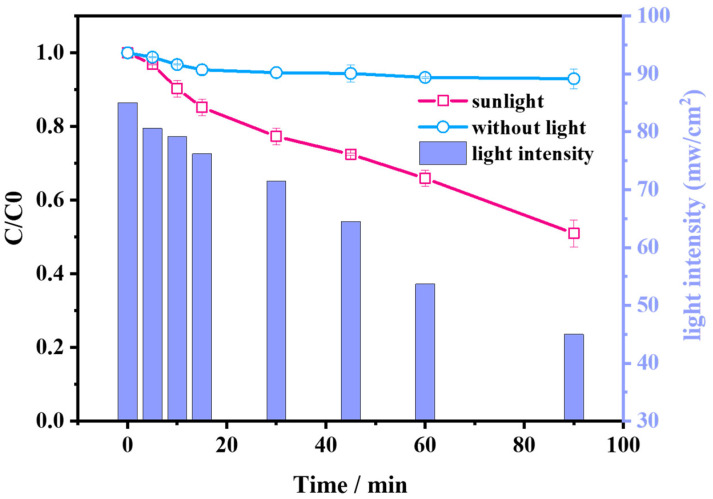
Degradation efficiency of AG in the presence or absence of sunlight and the change of light intensity during the test procedure.

Natural sunlight was considered as the light source to evaluate the efficacy of the AG/PMS self-catalytic system in order to verify the practical application potential of the proposed system. Degradation efficiency of AG in the presence or absence of sunlight as well as the change of light intensity during the test procedure are presented in Figure 3. The degradation ratio reached 50% after 90 min under natural sunlight irradiation, with the light intensity changing as the reaction proceeded, while the degradation ratio was only 8.0% when the system was sheltered from sunlight. This is because in the practical application system, apart from visible light illumination, the sunlight also contains 5% UV light, which can directly activate persulfate and thus enhance the activation process of persulfate [18,33]. Meanwhile, the natural sunlight utilized in the system is renewable and inexhaustible. Thus, the application of the self-catalytic system under natural sunlight irradiation can provide an energy-saving and sustainable pathway for the degradation of pollutants, which has a broad application prospect.

### 2.4. Mechanism of AG Self-Activating PMS

#### 2.4.1. Identification of Reactive Oxidizing Species

Quenching experiments and electron paramagnetic resonance (EPR) analysis were carried out to investigate the reactive oxidizing species generated during the reaction process in order to reveal the mechanism of AG self-activating PMS [34]. Generally, SO_4_^•−^, •OH, superoxide radical (O_2_^•−^) and singlet oxygen (^1^O_2_) are the dominant reactive oxidizing species involved in the PMS catalytic system. In the quenching experiments, methanol (MeOH) was introduced for quenching SO_4_^•−^ and •OH [35,36], meanwhile tert-butanol alcohol (TBA) could be specifically used for quenching •OH because of the higher reaction rate constant (k = 3.8~7.6 × 10^8^ M^−1^s^−1^) compared to capturing SO_4_^•−^ (k = 4.0~9.1 × 10^5^ M^−1^s^−1^) [37,38,39]. In addition, furfuryl alcohol (FFA) was commonly employed as the ^1^O_2_ quencher [29].

**Figure 4:**
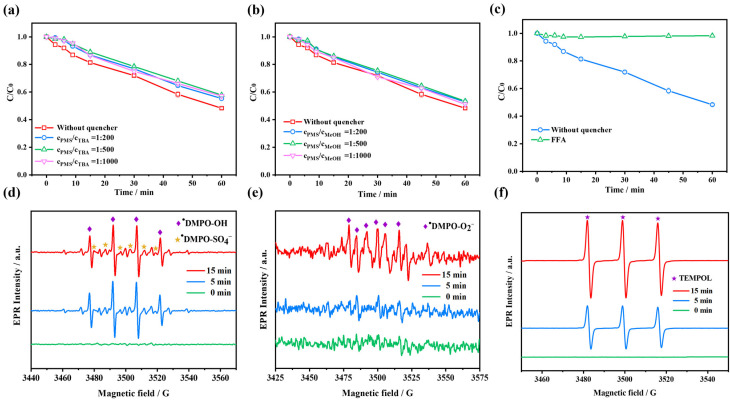
Effects of (**a**) methanol (MeOH), (**b**) tert-butanol alcohol (TBA) and (**c**) furfuryl alcohol (FFA) on AG degradation; EPR signals of (**d**) ^•^DMPO (5,5-dimethyl-1-pyrroline oxide)-SO_4_^−^ and ^•^DMPO-OH, (**e**) ^•^DMPO-O_2_^−^ and (**f**) TEMPOL (2,2,6,6-tetramethylpiperidine-1-oxyl) adducts at different reaction times in the AG/PMS self-catalytic system. (Reaction conditions: initial pH = 7.33, [AG]_0_ = 10 mg/L, [PMS]_0_ = 1 mM, [DMPO]_0_ or [TEMP (2,2,6,6-tetramethyl-4-piperidinol)]_0_ = 100 mM; EPR spectrometer settings: microwave frequency, 9.86 GHz; microwave power, 20 mW; center field, 3520 G; gain, 2.0 × 10^5^; modulation amplitude, 10 G; scan, 60 s; number of scans: 3).

Different quencher concentrations were set up to evaluate the influence on the degradation of AG. Figure 4a–c represents the concentration of the reagent. The degradation ratio of AG dropped from 52.2% to roughly 46% at each of the three concentrations after MeOH quenching, as shown in Figure 4a, which means there are at least one of •OH and SO_4_^•−^ in the catalytic system, probably in small amounts. Similarly, the dye degradation ratio was reduced to about 42% at all three concentrations in the TBA quenching experiments (Figure 4b), which indicates there might be a small amount of •OH in the system. Note that, in general, MeOH has a stronger inhibition effect on the degradation of pollutants than TBA since it can quench both •OH and SO_4_^•−^. However, from the above experimental results, it is evident that the TBA system has a slightly stronger inhibition compared to the MeOH system, which probably results from the fact that TBA can hinder the electron transfer process within the system [40,41]. Therefore, the presence of •OH and SO_4_^•−^ need to be further verified in combination with EPR experiments due to their small amounts. In contrast, with the addition of 10 mM FFA, the degradation ratio of AG dropped dramatically from 52.2% to 1.77% (Figure 4c), with a significant inhibition process, indicating that ^1^O_2_ plays a very important role in the system.

Additionally, in situ EPR analysis was employed to further verify the reactive oxidizing species produced during this system. As shown in Figure 4d, signals of ^•^DMPO (5,5-dimethyl-1-pyrroline oxide)-OH (a_N_ = a_H_ = 14.8 G, g = 2.0058) [42] and ^•^DMPO-SO_4_^−^ (a_N_ = 13.51 G, a_H_ = 9.93 G, a_H_ =1.34 G, a_H_ =0.88 G, g = 2.0058) [43] can be detected, which implies the presence of •OH and SO_4_^•−^ in the system. Meanwhile, a weak signal of ^•^DMPO-O_2_^−^ with aN = 13.0 G, aH = 10.2 G, aH =1.3 G and g = 2.0059 was also detected in the MeOH medium after 15 min of reaction, implying the possible presence of O_2_^•−^ in the system. The reason for O_2_^•−^ captured in the MeOH medium is that O_2_^•−^ is unstable in the aqueous medium and reacts rapidly to produce other species, such as H_2_O_2_ and ^1^O_2_ [44,45,46]. Furthermore, the significant 2,2,6,6-tetramethylpiperidine-1-oxyl (TEMPOL) signal (a_N_ = 16.9 G, g = 2.0058) [42] through the dominant oxidation by ^1^O_2_ appeared as depicted in Figure 4f, indicating that there may be massive ^1^O_2_ in this catalytic system.

Combined with quenching experiments and EPR analysis results, it can be concluded that the degradation of AG is achieved through a combination of radical and non-radical pathways, where ^1^O_2_ plays a major role while •OH, O_2_^•−^ and SO_4_^•−^ are secondary.

#### 2.4.2. Characterization of Electron Transfer Processes

**Figure 5:**
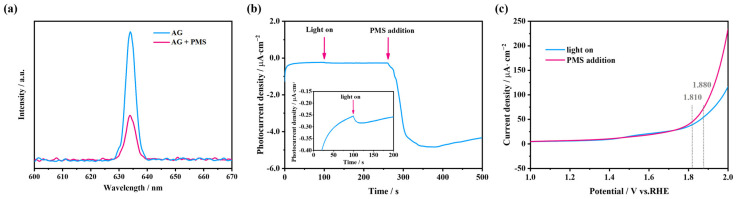
(**a**) Changes in fluorescence intensity of AG before and after PMS addition; (**b**) chronoamperometry analysis of the AG/PMS self-catalytic system; (**c**) linear sweep voltammetry (LSV) curves under visible-light irradiation and PMS addition.

Previously, we have elucidated how the dye-sensitized photocatalytic system works. When the dye molecules are irradiated by visible light, the photo-induced electrons inside the highest occupied molecular orbital (HOMO) jump to its lowest unoccupied molecular orbitals (LUMO), then are injected in the conduction band of the semiconductor, ultimately being captured by oxidants or dissolved oxygen [47]. In the AG/PMS self-catalytic system, we deduce that the reaction is similar to the aforementioned process, in which the photo-induced electrons in the LUMO of AG are directly captured by PMS. To verify this conjecture, firstly, we employed photoluminescence spectra to depict the electron transfer process. Generally speaking, the photo-induced electron–hole pairs recombination process of organics or catalysts generates fluorescence, and the high intensity of fluorescence is largely related to the excessive recombination [48]. On the contrary, lower intensity of fluorescence indicates a stronger ability of electron–hole pairs to separate [49]. Change in fluorescence intensity before and after PMS addition into the photo-excited AG solution is shown in Figure 5a. The obvious decrease in the fluorescence intensity appeared after PMS was added in, and based on the above principle, it can be illustrated that the photo-excited AG can effectively transfer photo-induced electrons to PMS.

Moreover, to further verify the electron transfer process from the photo-excited dye to PMS, electrochemical analysis of chronoamperometry and linear sweep voltammetry (LSV) was performed. As shown in Figure 5b, when the light was turned on, the transient current intensity changed, indicating that the AG loaded on the glassy carbon electrode generates photo-induced electrons when irradiated by visible light. Furthermore, the current density of the AG/PMS self-catalytic system raised swiftly from 0.410 to 4.841 μA/cm^2^ after PMS addition, suggesting the occurrence of an efficient electron transfer process, and the reverse current indicates that the electrons swiftly migrate from the photo-excited dye to PMS [50]. Moreover, the LSV of the samples was also measured under visible-light irradiation after PMS addition. As depicted in Figure 5c, after adding PMS, the current density was significantly higher than that when irradiated with visible light only, which is consistent with the chronoamperometry analysis. Concretely, the overpotential is 1.880 V when exposed to visible light only, whereas it decreases to 1.820 V after adding PMS, which is, respectively, due to the aggregation of photo-induced carriers and the transfer of large number of separated electrons from photo-excited dyes to PMS molecules [51]. Based on the above analysis, it can be stated that in the self-catalytic system, the photo-excited AG dye can effectively transfer photo-induced electrons to PMS, which is the foundation of the subsequent PMS activation and dye degradation process.

#### 2.4.3. Degradation Route of AG

**Figure 6:**
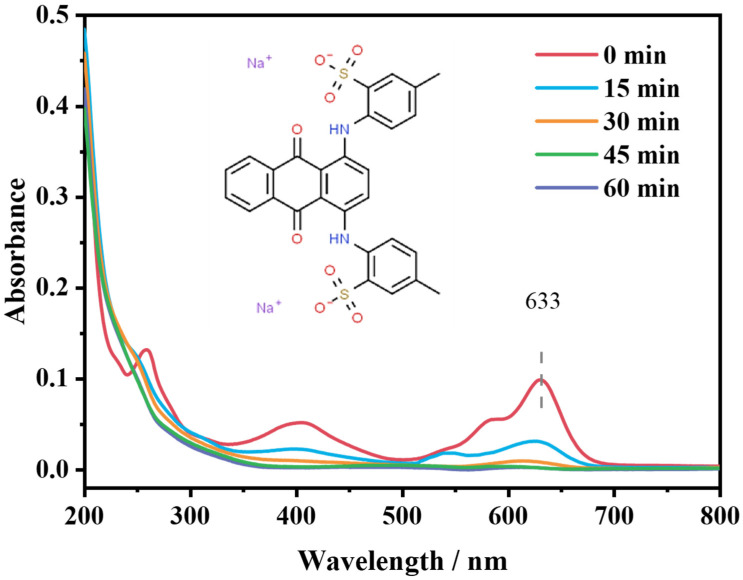
Changes in UV-vis spectra for different reaction times.

During the degradation process of pollutants, their molecular structures change over time, which leads to variation in the location and intensity of their UV-vis absorption spectral peaks [52]. In this paper, UV-vis absorption spectra were performed in the range of 200–800 nm for solutions in the reaction at 0, 15, 30, 45 and 60 min of AG (10 mg/L) self-activating PMS experiments under light conditions. As shown in Figure 6, the UV-Vis absorption of AG gradually decreased as the reaction proceeded, implying that the large conjugated system, anthraquinone ring and benzene ring in the structure of AG were steadily degrading [7,53]. Throughout the reaction, the color of the AG dye solution gradually becomes lighter until it is colorless.

**Figure 7:**
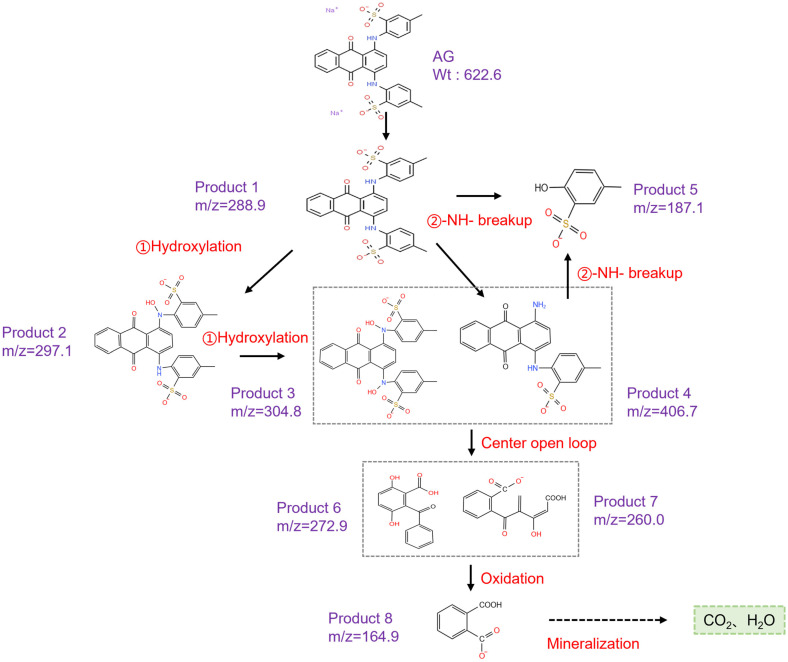
Possible degradation pathways of AG on self-activating PMS.

To further elucidate the degradation pathways of AG on its self-activating PMS, the generated intermediates were verified using Ultra Performance Liquid Chromatography-Quadrupole-Time of Flight Mass Spectrometer. Based on the obtained mass spectra of the intermediates (Figures S1 and S2) and previous studies [54,55], the possible AG degradation pathways are proposed in Figure 7. The molecular weight of AG is 622.6, and a strong peak, with *m*/*z* = 288.9, was observed at the beginning of reaction, which may be due to the dissociation of Na^+^ on the two -SO_3_ groups of the AG molecule in aqueous solution, corresponding to the structure [AG-2Na]^2−^. There are two possible degradation pathways for [AG-2Na]^2−^, and one is the hydroxylation process, which generates product 2 and product 3, with peaks at *m*/*z* = 297.1 and 304.8, respectively. The other is the breakup of the -NH- group to produce product 4 (*m*/*z* = 406.7) and product 5 (*m*/*z* = 187.1), which may lead to the disappearance of the absorption peak at around 633 nm in Figure 6. Products 3 and 4 generated by the two degradation pathways undergo central ring opening to generate product 6 (*m*/*z* = 272.9) and product 7 (*m*/*z* = 260.0), followed by a continuous attack of the reactive oxidizing species to generate phthalic acid (product 8, *m*/*z* = 164.9), and finally the intermediate products are gradually mineralized to CO_2_ and H_2_O.

#### 2.4.4. Mechanism of AG Self-Activating PMS

**Figure 8:**
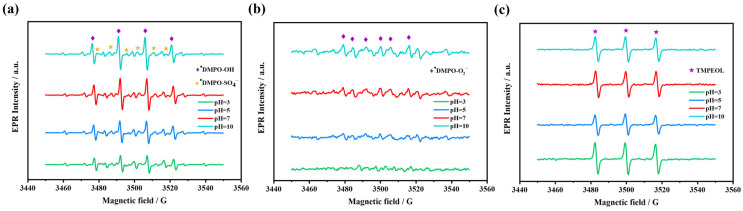
EPR signals of (**a**) ^•^DMPO-SO_4_^−^ and ^•^DMPO-OH; (**b**) ^•^DMPO-O_2_^−^; (**c**) TEMPOL adducts in the AG/PMS self-catalytic system under different pH conditions (Reaction conditions: [AG]_0_ = 10 mg/L, [PMS]_0_ = 1 mM, [DMPO]_0_ or [TEMP]_0_ = 100 mM, reaction time: 15 min; EPR spectrometer settings: microwave frequency, 9.86 GHz; microwave power, 20 mW; center field, 3520 G; gain, 2.0 × 10^5^; modulation amplitude, 10 G; scan, 60 s; number of scans: 3).

According to the chemical quenching experiments and EPR analysis, it was demonstrated that the degradation of AG occurs mainly based on the reactive oxidizing species dominated by ^1^O_2_, and supplemented by •OH, O_2_^•−^ and SO_4_^•−^ in the photocatalytic system when pH is 7.33. However, pH may affect the type and content of the reactive oxidizing species, leading to different activation mechanisms. Therefore, the reactive oxidizing species under different pH conditions were further investigated using the EPR analysis, which was performed in the same way as in Section 2.4.1. The experiments were set up with four initial pH gradients of 3, 5, 7 and 10. The signals of ^•^DMPO-OH (a_N_ = a_H_ = 14.8 G, g = 2.0058) and ^•^DMPO-SO_4_^−^ (a_N_ = 13.51 G, a_H_ = 9.93 G, a_H_ = 1.34 G, a_H_ = 0.88 G, g = 2.0058) were detected at all pH conditions (Figure 8a). Additionally, the signal intensity of ^•^DMPO-OH increased with the rise of pH, the reason for which, on one hand, may be attributed to that a small amount of SO_4_^•−^ in the system that can be converted to •OH especially under alkaline conditions (Equation (6) or (7)) [56]; on the other hand, DMPO is unstable at pH less than 6.0 due to its pKa of 6.0 and will be protonated to form DMPO-H^+^, which leads to its instability to react with •OH or O_2_^•−^ [57,58] Therefore, the weak signal of ^•^DMPO-OH adduct under acidic conditions is not only related to the reduced concentration of OH^−^, but also to the instability of DMPO and its adducts under acidic conditions [42,59]. Meanwhile, the signal of ^•^DMPO-O_2_^−^ (aN = 13.0 G, aH = 10.2 G, aH =1.3 G, g = 2.0059) also showed the tendency to enhance with pH (Figure 8b), which is also due to the instability of DMPO under acidic conditions. In addition, the characteristic 1:1:1 triplet signal of TEMPOL is observed in the EPR spectra at all pH conditions (Figure 8c), indicating the possible presence of ^1^O_2_ in the AG/PMS self-catalytic system. The strongest signal of TEMPOL at pH = 3 is consistent with the effect of pH on the AG/PMS self-catalytic system, which again confirms the dominant oxidative degradation role of ^1^O_2_ in the degradation of AG. 

**Figure 9:**
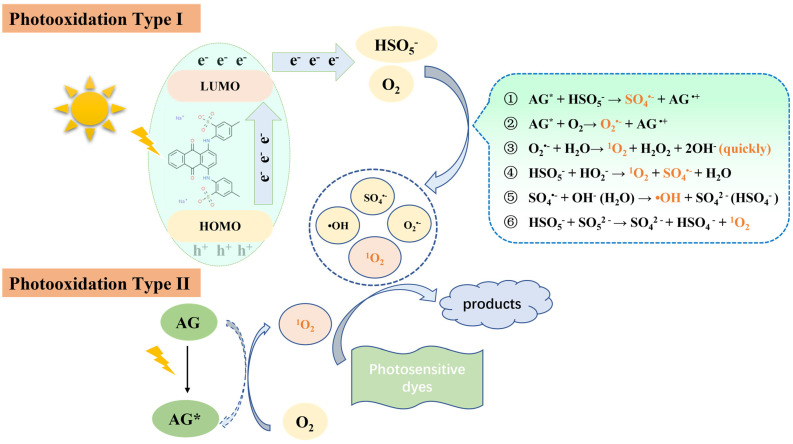
Mechanism diagram of AG self-activating PMS.

According to the obtained results, the possible mechanism of AG self-activating PMS is proposed (Figure 9). In general, the photooxidation process of photosensitive substances occurs through two mechanisms: (1) electron transfer (type I photooxidation); (2) energy transfer (type II photooxidation) [60,61]. In the type I photooxidation, AG transforms from the ground state to the excited state (AG*) when irradiated by visible light within its light absorption range (Equation (3)) [18,62], and the outer electrons in its HOMO jump into the LUMO. Except for the inevitable recombination of electron–hole pairs, part of photo-induced electrons are captured by HSO_5_^−^ to produce SO_4_^•−^ (Equation (4)) [43,63]. The remaining electrons are captured by O_2_ to generate O_2_^•−^ (Equation (5)) [61], but they rapidly react with H_2_O to produce ^1^O_2_ (Equation (6)). Meanwhile, the other HSO_5_^−^ reacts with HO_2_^−^ to produce ^1^O_2_ and SO_4_^•−^ (Equation (7)), and most of the SO_4_^•−^ produced via Equations (4) and (7) reacts with OH^−^ or H_2_O to form •OH (Equation (8) or (9)), depending on the pH of the reaction solution, which accounts for the small amount of SO_4_^•−^ in the system.

During type II photooxidation, the energy transfer process from AG* to O_2_ could result in the production of ^1^O_2_ (Equation (10)) [43,64]. In addition, the self-oxidation of PMS also contributes to the production of ^1^O_2_ [65,66] (Equation (11)). In conclusion, it is mainly the ^1^O_2_ produced via Equations (6), (7), (10) and (11), the •OH produced via Equation (8) or (9) as well as the remaining SO_4_^•−^ and O_2_^•−^ that contribute to the degradation of AG (Equation (12)). Eventually, AG dyes are continuously attacked by these reactive oxidizing species and undergo the hydroxylation process, the breakup of -NH- group, central ring opening, and finally the intermediate products are gradually mineralized to CO_2_ and H_2_O. Herein, the mechanism of the reactive oxidizing species production and the route of AG degradation are described below:

(3) $$\mathrm{AG}\;+\;h\nu \;\to {\;\mathrm{AG}}^{*}$$

Photooxidation Type I: electron transfer

(4) $${\mathrm{AG}}^{*}+{\;\mathrm{HSO}}_{5}{}^{-}\;\to \;{\mathrm{AG}}^{•+}+{\;\mathrm{SO}}_{4}{}^{•-}\;$$

(5) $${\mathrm{AG}}^{*}+{\;O}_{2}\to {\;O}_{2}{}^{•-}+{\mathrm{AG}}^{•+}$$

(6) O2•−+ H2O→ O21+H2O2+2OH−

(7) HSO5−+ HO2− → O21+ SO4•−+ H2O 

(8) $${\mathrm{SO}}_{4}{}^{•-}+{\;\mathrm{OH}}^{-}\;\to \;•\mathrm{OH}\;+{\;\mathrm{SO}}_{4}{}^{2-}$$

(9) $${\;\mathrm{SO}}_{4}{}^{•-}+{\;H}_{2}O\to \;•\mathrm{OH}\;+{\;\mathrm{HSO}}_{4}{}^{-}$$

Photooxidation Type II: energy transfer

(10) AG*+ O2 →O21+ AG

(11) HSO5−+ SO52− → SO42−+ HSO4−+O21

(12) O21 /•OH/O2•−/SO4•−/+ AG → products

## 3. Materials and Methods

### 3.1. Chemicals

Alizarin green, ethanol, methanol (MeOH), tert-butanol alcohol (TBA) and furfuryl alcohol (FFA) were all purchased from Macklin Biochemical Co., Ltd. (Shanghai, China). Potassium peroxymonosulfate (PMS), 5,5-dimethyl-1-pyrroline oxide (DMPO), 2,2,6,6-tetramethyl-4-piperidinol (TEMP) and Nafion solution were purchased from Aladdin Chemistry Co., Ltd. (Shanghai, China). Sodium sulfate (Na_2_SO_4_) was purchased from Tianjin kemiou Chemical Reagent Co., Ltd. (Tianjin, China). The experimental water was all 18.25 MΩ·cm ultrapure water, provided by the Milli-Q gradient ultrapure water system.

### 3.2. Experimental Procedures

The experiments were carried out in a dark chamber in which the light source was a 300 W xenon lamp (CEL-HXF300, Beijing China Education Au-light Co., Ltd., Beijing, China) with a 400 nm cut-off filter, and the general light intensity was 100 mW/cm^2^. The reaction solution was a mixture of 100 mL 10 mg/L AG and 1 mM PMS. Before switching on the light source, PMS was added in, and then the mixture was taken at fixed time intervals to detect the change in AG concentration. Moreover, the experiments under natural sunlight irradiation were conducted outdoors at noon in summer, and the 100 mL mixture of 10 mg/L AG and 1 mM PMS was exposed to sunlight for 90 min. Samples were obtained at regular time intervals to examine the change in dye concentration.

### 3.3. Analytical Methods

The concentration of AG dye was detected at λ = 633 nm using an ultraviolet-visible spectrophotometer (UV-4802, Shanghai Unico Instruments Co., Ltd., Shanghai, China). Changes in fluorescence intensity were detected using a fluorescence spectrometer (Edinburgh FLS9, Germany). Light intensity was recorded with a datalogging solar power meter (TES-1333R). The decomposition efficiency of PMS was obtained using the iodometric method [20]. It was specifically performed by mixing 0.5 mL of a sample with 2.0 mL 10 g/L of NaHCO_3_ and 2.5 mL 166 g/L of KI, then after complete shaking, the mixture was allowed to keep stationary for 20 min and measured at λ = 352 nm using ultraviolet-visible spectrophotometer (UV-4802, Shanghai Unico Instruments Co., Ltd.).

The electron transfer process between photosensitive dye and PMS was characterized using an electrochemical workstation (CHI660E, Shanghai Chenhua) in 0.05 mM Na_2_SO_4_ solution with the three-electrode system, in which glassy carbon electrode (GCE) with AG was employed as the working electrode and Pt acted as the counter electrode. As for the reference electrodes, saturated calomel electrodes for photocurrent tests and Ag/AgCl electrode for linear scanning voltammetry tests were employed, respectively. Note that the working electrode was prepared by adding 10 μL of catalyst ink dropwise to the glassy carbon electrode, where the homogeneous catalyst ink was formed by dispersing 4 mg of AG dye into 800 μL of ethanol and 200 μL of a 5 wt% Nafion solution.

Identification and characterization of the main reactive oxidizing species in different reaction systems were performed using an electron paramagnetic resonance spectrometer (EPR, Bruke ZMXmicro-6/1, Germany) in situ. A well-known spin trapping agent for •OH, SO_4_^•−^ and O_2_^•−^ is DMPO, which functioned in different solvent media, where •OH and SO_4_^•−^ was captured in ultrapure water, while O_2_^•−^ was trapped in methanol [24]. Moreover, TEMP was used to support the generation of ^1^O_2_, since TEMP would generate typical three-linear EPR signals of TEMPOL through the dominant oxidation by ^1^O_2_ [43,67,68]. The specific steps of the EPR experiments are as follows: before starting the experiment, 1 M DMPO and 0.5 M TEMP solutions were pre-configured, and then 50 μL DMPO (or 100 μL TEMP) was packed in a 2.0 mL brown centrifuge tube. After the catalytic reaction was started, samples were taken at different times using a syringe and filtered through a 0.45 μm membrane. Then, 0.5 mL of the filtered reaction solution was quickly added to the corresponding centrifuge tube and shaken well, after which the mixture was taken using a 50 μL capillary tube for EPR analysis. 

The solutions at different reaction times were tested using Ultra Performance Liquid Chromatography-Quadrupole-Time of Flight Mass Spectrometer (I-Class VION IMS Qtof, WATERS) for the analysis of the AG degradation products. Parameters: Column: ACQUITY UPLC^®^BEH C18 (1.7 μm, 100 mm × 2.1 mm); mobile phase: 0.1% formic acid: acetonitrile (70: 30); flow rate: 0.15 mL/min; column temperature: 25 °C; and injection volume: 10 μL. Ion source: ESI source; scanning mode: ESI negative ion mode; capillary voltage: 2.5 kV; ion source temperature: 100 °C; desolvation gas flow: 600 L/h; collision energy: 6–45 V; scan time: 0.2 s; and mass scan range *m*/*z*: 50–2000.

## 4. Conclusions

In this paper, a novel catalytic system of photo-excited organic dyes self-activating PMS without catalyst addition was proposed, in which the photo-excited dyes were self-degraded simultaneously as pollutants. Herein, under visible-light irradiation, 52.2% of 10 mg/L AG was degraded during 60 min with 1 mM PMS without catalyst addition, and it was found that the higher PMS dosage, the more acidic pH condition and subsequently the stronger light intensity contribute to the higher efficiency of dye degradation. The photo-excited AG can effectively transfer electrons to PMS for its activation, resulting in the generation of reactive oxidizing species dominated by ^1^O_2_, and supplemented by •OH, O_2_^•−^ and SO_4_^•−^, which further triggers the degradation of the dye activator. In addition, this system has the potential for practical application; in particular, the reaction could be carried out under natural sunlight irradiation, which could result in significant energy savings with respect to other activation methods for PMS. Therefore, the construction of photosensitive dyes/PMS self-catalytic system provides a novel idea of “using waste to treat waste” for dyeing wastewater, which has great significance in developing and improving the wastewater treatment process in a high-efficiency and low-consumption way.

## Data Availability

Not applicable.

## Supplementary Materials

The following supporting information can be downloaded at: https://www.mdpi.com/article/10.3390/molecules28104237/s1, Figure S1: Mass spectra of alizarin green degradation products 1 to 4; Figure S2: Mass spectra of alizarin green degradation products 5 to 8.

## Sample Availability

Samples of the compounds are available from the authors.
