# Supplementary material for: Photosensitive Dye as an Ideal Peroxymonosulfate Activator for Efficient Self-Degradation: A Novel Idea of Using Waste to Treat Waste
Source: Molecules. 2023 May 22;28(10):4237. doi: 10.3390/molecules28104237 (PMC10222008; doi:10.3390/molecules28104237)
Supplement: Supplementary file 1 [file molecules-28-04237-s001.zip › molecules-2222310-supplementary.pdf]

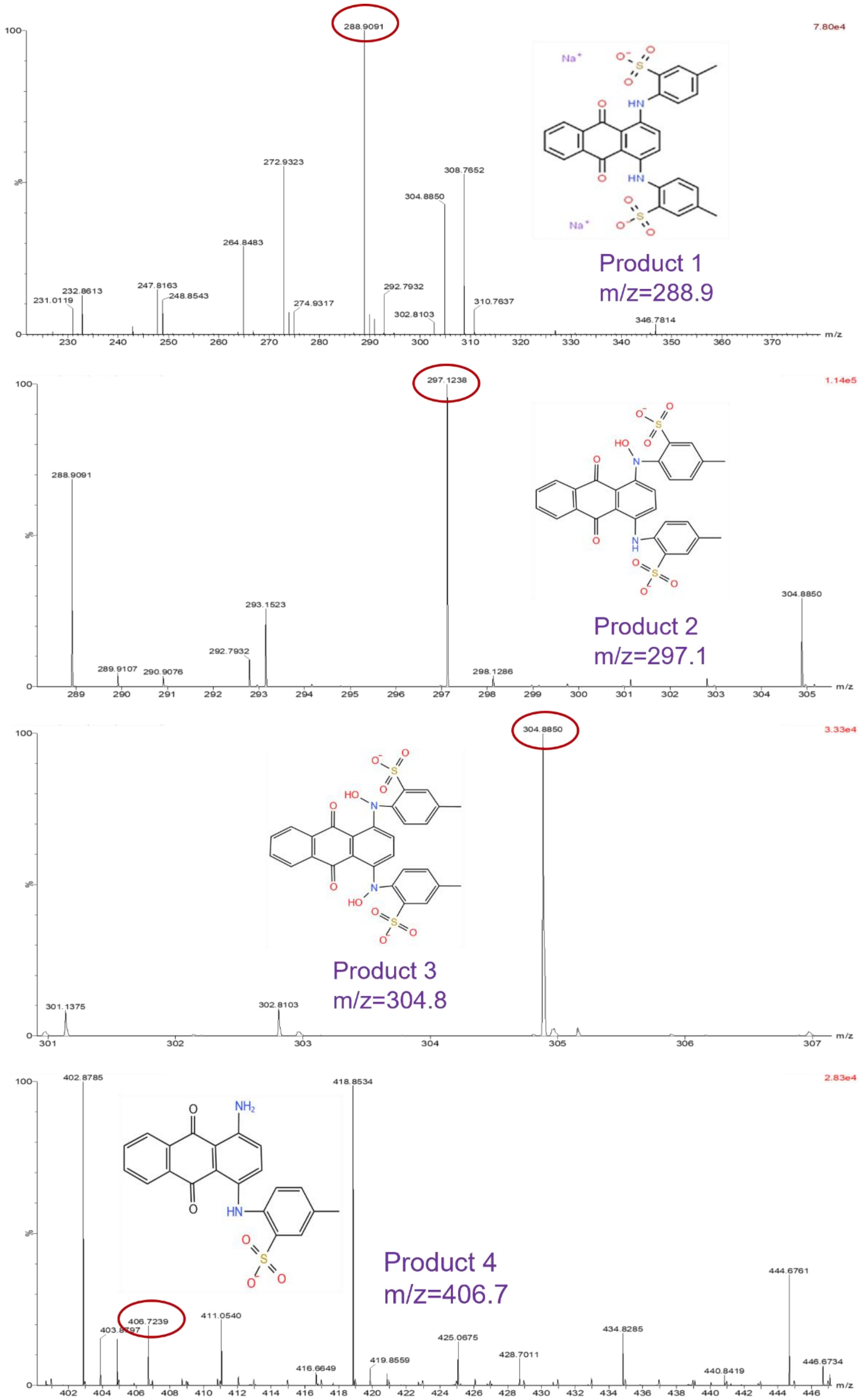

Figure S1: Mass spectra of alizarin green degradation products 1 to 4

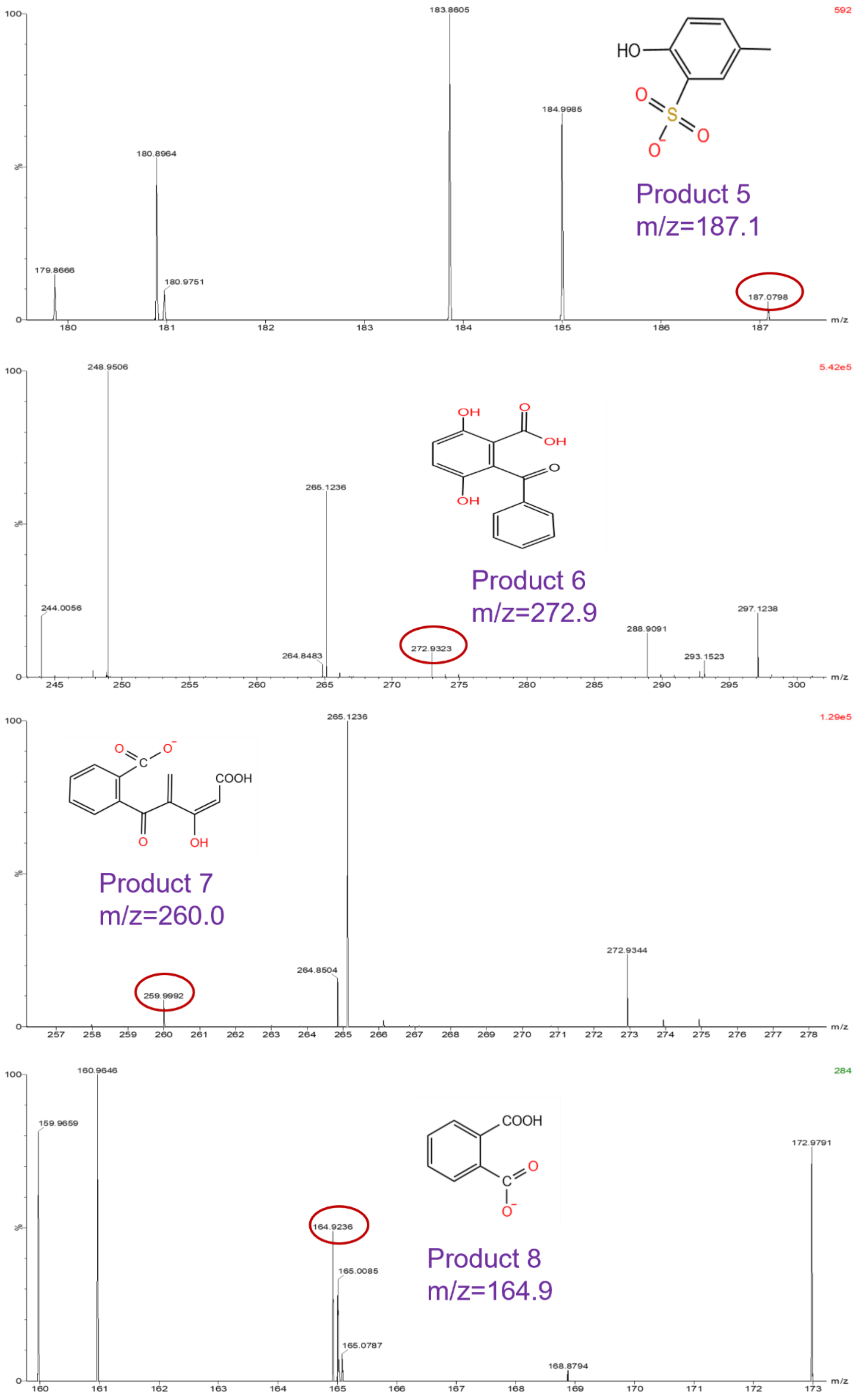

Figure S2: Mass spectra of alizarin green degradation products 5 to 8
